# The effectiveness of a community-based video-facilitated parenting intervention for child development integrated into routine maternal and child care services in India

**DOI:** 10.1371/journal.pgph.0005434

**Published:** 2026-03-02

**Authors:** Reetabrata Roy, Aanchal Chopra, Gitanjali Lall, Deepak Jangra, Madhavilatha Maganti, Vikram Patel, Gauri Divan

**Affiliations:** 1 Child Development Group, Sangath, Bhatkar Waddo, Socorro, Bardez, Porvorim, Goa, India; 2 Department of Health Promotion, Education and Behaviour, Arnold School of Public Health, University of South Carolina, Columbia, South Carolina, United States of America; 3 Division of Sciences, School of Interwoven Arts and Sciences (SIAS), Krea University, Sri City, Andhra Pradesh, India; 4 Ashoka University, Sonipat, Haryana, India; 5 Department of Global Health and Social Medicine, Harvard Medical School, Boston, Massachusetts, United States of America; DTHF: Desmond Tutu HIV Foundation, SOUTH AFRICA

## Abstract

Parenting interventions integrating nutrition, stimulation, and responsive caregiving promote child development but face challenges to scale in low-resource settings. This study describes implementation and effectiveness of *Aalana Palana*, a facilitated video-viewing intervention on responsive parenting, embedded in routine maternal and child care services in India. *Aalana Palana* was delivered by Anganwadi workers (AWWs) in Telangana, India. A quasi-experimental, non-randomized controlled design was used to evaluate its effectiveness. Intervention group were caregivers of children aged <3 years who reported seeing at least one intervention video in the three months preceding data collection; controls included caregivers who hadn’t seen videos in this period. The primary outcome was child development measured using the CREDI, a caregiver report of child’s development used extensively in LMICs. We analysed moderation by baseline socio-demographic characteristics and mediation of home environment on CREDI. 30 AWWs delivered *Aalana Palana* sessions; 361 sessions were observed by supervisors for quality assessment, reporting high fidelity. 1824 caregivers (91·5% eligible caregivers) gave consent. *Aalana Palana* improved overall child development (exposed: -0·58 (0·90), unexposed: -1·0 (0·92); β = 0·16, p < 0·001); improvements were observed across all CREDI domains. Home environment mediated the effect of the intervention (β = 0·027 (0·014,0·044)). Intervention effects were larger for children aged <1 year compared to older age groups. *Aalana Palana* is an effective strategy for scaling up parenting interventions for child development in low-resource settings. This study was funded by the UNICEF office of Andhra Pradesh, Karnataka and Telangana. Registration Number: NCT07004608.

## Introduction

Children have the best chance of reaching their developmental potential when caregivers and key stakeholders, especially community-based workers (CBWs) in their proximal environment, and policies in the distal context provide them with nurturing care [1]. This includes a home environment which is responsive and sensitive to children’s nutritional and health needs, provides opportunities for early learning, includes stimulating interactions with caregivers, and safeguards them from adversities [2]. The number of children below five years of age, at risk of not reaching their developmental potential decreased from 279·1 to 249·4 million between 2004 and 2010, across all income groups, particularly in South Asia. Early childhood development (ECD) interventions, specifically in the first 1000 days of life, which integrate nutrition and opportunities for play, show better impacts on child development and nutrition compared to nutritional interventions alone [3]. Also, parenting interventions that focus on responsive caregiving compared to those that do not, reported greater effects on child cognitive development, parenting knowledge and practices, and parent-child interactions [4].

While there is consensus to implement such parenting programs at scale, existing child care systems are inadequately resourced and overstretched [5] and may overburden existing CBWs and impact their performance if due attention is not given to their work environment [6]. Therefore, employing contextual innovations and empowering CBWs with a combination of relevant training, supportive supervision, technology, and adequate remuneration may facilitate intervention scale up [7] as CBWs act as a vital link between systems and communities [8]. One such strategy is the use of mobile health technologies, including interventions delivered through videos, which can reduce CBW workload and enhance the quality of community facing messaging provided by them [9]. Videos offer an efficient and feasible method of improving parent and child outcomes in low and middle income countries (LMICs) [10], being particularly effective in enhancing caregiver attention, engagement, and learning of new behaviours [11]. Moreover, video-based parent coaching, specifically video-viewing, is effective in making large-scale programs accessible in LMICs where staffing and resources are sub-optimal [12]. Video-based interventions have been used in LMICs to promote ECD knowledge, early stimulation, fathers’ engagement, household environment safety [13], child cognition and maternal knowledge of child development [14]. In this paper we describe the implementation and effectiveness of a scalable, facilitated video-viewing parenting intervention combining nutrition and responsive caregiving in Telangana, India.

**Research in context**

***Evidence before this study***

A nurturing environment in the first 1000 days of life, beginning from conception, are crucial for child development. Early childhood interventions targeting components of nurturing care, specifically nutrition, responsive parenting, and early learning opportunities improve child development and nutrition, parenting knowledge, and parent-child interactions. Given the under resourced and overburdened health systems in LMICs, innovations such as video-based interventions can not only reduce CBWs workload and improve fidelity of intervention delivery, but also enhance caregiver attention and learning, ECD knowledge and child cognitive development. However, evidence on the effectiveness of such interventions integrated in community maternal and child healthcare programs is limited.

***Added value of this study***

To our knowledge, this is the first real-world implementation study in a low resource setting evaluating a facilitated video-based intervention, *Aalana Palana*, integrated into existing systems. It was found that *Aalana Palana* was delivered with high levels of fidelity by system level CBWs and enhanced child development, particularly in the cognitive and socio-emotional domains. The intervention also improved home environment and feeding practices, and the effect of the intervention was mediated by changes in the home environment. We also observed a greater effect in younger children, consistent with development theory.

***Implications of all available evidence***

Evidence-based parenting interventions for promoting early child development can be successfully scaled up through routine maternal and child healthcare platforms with video-facilitation, alignment of training and supervision procedures with existing systems, and observation-based quality monitoring.

## Methods

### Ethics statement

Ethical approval was granted by the Sangath Institutional Review Board (Sangath Institutional Review Board is registered with the Office for Human Research Protections (OHRP) (IORG0002715) and Federal Wide Assurance (FWA) (FWA00004350) under the Department of Health and Human Services (HHS), USA.) on 03 January 2023, vide ref no: RR_2019_58. Written informed consent was obtained from all study participants.

### Study setting

Telangana, founded in 2014, is the youngest state in India. According to the National Family Health Survey-5 (NFHS-5, 2019–21) [15], 64·8% women in the state were literate, with a notable urban-rural difference (urban 78·6%, rural 56·6%). Infant mortality rate decreased slightly between NFHS-4 (2014–15) and NFHS-5 from 27·7 to 26·4; similar trends were noted for under-five (U5) mortality which decreased from 31·7 to 29·4. Maternal and child care indicators for the state were better than national averages – 70·4% mothers received at least four antenatal care visits, 97% births were institutional, and 84·7% children aged 12–23 months were fully vaccinated. However, child feeding practice indicators showed that only 37·1% children under three months were breastfed within one hour of birth, 68·2% children under six months were exclusively breastfed, 51·3% children aged 6–8 months received solid or semi-solid food along with breast milk and only 9·2% children aged 6–23 months received adequate diet. Also, there was a marked worsening of U5 child anthropometry indicators between NFHS-4 and 5, where stunting went up from 28% to 33·1%, underweight from 28·4% to 31·8% and wasting from 18·1% to 21·7%. Early childhood undernutrition is likely to be influenced by a range of infant and young child feeding (IYCF) practices related to maternal, socioeconomic, and demographic factors, with higher vulnerability observed among younger and less-educated mothers, and low-income households; conversely, higher maternal socioeconomic status and adequate antenatal care are associated with improved IYCF practices [16,17].

While these child health indicators highlighted the need to develop effective ECD interventions for Telangana, high mobile ownership in the state (96% urban and 91% rural households having a mobile phone, and 60% women having access to phones they use themselves [15]) provided an opportunity to deliver a facilitated video-viewing intervention.

This project was implemented between 2019–2023 by the NGO Sangath (www.sangath.in) in collaboration with United Nations Children’s Fund (UNICEF) and Department of Women Development and Child Welfare (DWDCW; Government of Telangana, India). We embedded a facilitated video-viewing intervention called *Aalana Palana* (‘caring and nurturing’ in the local language Telugu) within 30 Anganwadi Centres (AWCs) of the Integrated Child Development Services (ICDS) Scheme in Telangana. AWCs are community creches managed by CBWs called Anganwadi Workers (AWWs).

### The Aalana Palana intervention

The goal of *Aalana Palana* was to transform the delivery of messaging on responsive parenting and nutrition from a largely paper-based counselling approach to facilitated video-viewing, discussion and demonstration model, ensuring fidelity of intervention delivery by standardising content. AWWs used these videos in individual and group counselling sessions with caregivers at AWCs and family homes. *Aalana Palana* was developed using the following steps:

#### Development of video content.

Information synthesis: To develop content we reviewed parenting interventions specifically in LMICs, the Nurturing Care Framework, the WHO Care for Development Package, and national resources specifically from Telangana [18].

Blueprint: The resulting content was triangulated with findings from our formative research [19], and consultations with system-level stakeholders to develop blue-prints for key messages. Eleven videos were prioritized for development. ECD messages and parent-child interaction activities to be included in each video were listed to build a cohesive narrative for specific age groups from prenatal to 24 months and one additional video for 25–60 months.

Scripting: This blueprint guided the iterative development of scripts drafted in English which were reviewed by experts from Sangath, DWDCW and UNICEF. The scripts were then translated in Telugu and Hindi using colloquial language, culturally relevant and relatable terms. The scripts incorporated learning objectives, visuals, narration, and discussion points.

Video content development: A creative team shot content in local communities with pregnant women, caregivers and young children. These families were selected by AWWs based on briefings provided by Sangath team and consented before shoots. Concerted efforts were made to build rapport with families to ensure that the videos reflected real life interactions. For play activities, existing reference videos were shown and coaching was given to caregivers. We ensured equitable representation by including families and caregivers of both male and female children, belonging to varied social and religious groups.

Feedback on video content: Stakeholders from DWDCW, UNICEF and Sangath team members who had considerable experience of developing, implementing and testing parenting intervention focusing on the early years of life, iteratively provided feedback on the content. UNICEF team members also provided inputs based on their learnings from systemic engagement with the ICDS and the communities in Telangana for which the intervention was developed. During this process, language was further simplified and messaging was aligned to complement existing child development initiatives implemented within the state. Recommendations on organization of visuals within videos, language, voice modulation, and cultural diversity of content were used to refine the videos. S1 Text provides details of key messages for each *Aalana Palana* video.

#### Implementation of Aalana Palana.

Content and delivery of *Aalana Palana*: The videos promote responsive and sensitive parenting, early learning opportunities and desired nutritional practices for young children and pregnant women. The content describes ‘WHAT’ behaviours caregiver should adopt, ‘HOW’ they could include these behaviours in their routines and ‘WHY’ a particular behaviour is important for their child’s development. Each 5–6-minute video is divided into three segments. At the end of each segment instructions are displayed to pause and discuss key issues facilitating communication and sharing of opinions and concerns regarding viewed content. At the end of each video, a *sutradhaar* (narrator), summarizes key takeaways and actionable practices.

To support problem solving, 40 shorter 1–2-minute variants of these videos were developed and shared with caregivers who brought up specific challenges during sessions. These were developed concurrently during the implementation of *Aalana Palana*. For instance, when mothers shared concerns around their inability to give time to the child amidst busy household responsibilities, a short video demonstrated how they could engage with the child while doing their chores.

Training: In consultations with DWDCW and UNICEF we designed a two-day training which would be integrated within existing AWW training schedules. The content focused on experiential learning and role plays. Topics included the importance of early years, responsive caregiving, the *Aalana Palana* content and delivery processes, and supportive supervision. We uploaded the *Aalana Palana* videos onto the phones of AWWs using external solid-state drives and trained them to select age-specific videos for session delivery. 30 AWWs from three ICDS projects, along with their supervisors and Child Development Project Officers (CDPOs), were trained by Sangath, UNICEF, and DWDCW teams in December 2021.

Session delivery: Formative research findings highlighted challenges on time use and workload of AWWs [19], which informed the decision of prioritising group over individual counselling sessions. AWWs started delivering *Aalana Palana* sessions from January, 2022.

Supportive supervision: We conducted at least one in-person supervision visit per month for all 30 AWWs commencing in January 2022. For each visit the team used a 41 item semi-structured observation checklist (35 close ended and 6 opened items) to assess session quality. The checklist included items on session characteristics (viz., one-to-one or group delivery, session attendance and time take), presentation and navigation through the video content and the quality of sessions with focus on communications, discussions and demonstrations (see Table 1).

**Table 1:** Implementation results (N = 361).

Session Characteristics		% (n)
Type of session	One-to-one session	40·4 (146)
	One-to-group session	59·6 (215)
Mean attendees in group sessions (SD, Range) (Group session only; n = 215)		4·0 (1·7, 2-11)
Approximate session delivery time	10-15 mins	18·3 (66)
	16-20 mins	35·4 (128)
	21-25 mins	26·9 (97)
	26-30 + mins	19·4 (70)
Presentation & Navigation of videos		
Seated so that session attendees could see mobile screen		97·5 (352)
Navigated phone to find appropriate video for session		93·6 (338)
Checked all session attendees could see video		95·8 (346)
Checked all session attendees could hear video		96·1 (347)
Paused/played videos as required		83·9 (303)
Quality of sessions		
Verbal & non-verbal communication	Greeted caregiver(s) & made routine enquiries	94·2 (340)
	Used empathetic body language during session	98·6 (356)
	Asked open ended questions to caregiver(s)	87·0 (314)
Enlisting family support	Gave caregiver(s) space to respond	90·3 (326)
	Involve family member in the discussion	40·4 (146)
	Encourage caregiver to discuss about video with family member	62·9 (227)
Appropriate use of ‘*Aalana-Palana’* videos	Explained *Aalana-Palana*	81. 7 (295)
	Identified appropriate video for the session	97·5 (352)
	Was adequately fluent with the content of the video	70·1 (253)
	Summarized all messages of video at the end of session	45·7 (165)
	Shared video with caregiver on WhatsApp	60·7 (219)
Demonstration & Scaffolding	Demonstrated nutrition related activities	8·3 (30)
	Demonstrated parent-child interaction activities	18·0 (65)
	Guided caregiver(s) to do activities shown in video	35·5 (128)
	Asked caregivers to demonstrate activities shown in videos (Group session only; n = 215)	14·9 (32)
Discussion & problem solving	Paused & checked if caregiver(s) understood messages	81·2 (293)
	Asked caregiver’s opinion on videos	81·4 (294)
	Paused video & initiated discussions when required	77·6 (280)
	Enquired about challenges caregiver(s) may be facing	75·6 (273)
	Addressed concerns on child development	80·9 (292)
	Was able to respond queries raised by caregiver(s)	67·3 (243)
	Used positive gestures when the caregiver(s) said something right	52·9 (191)
	Made sure that caregivers participated equally (Group session only; n = 215)	54·4(117)
	Initiate & moderate discussions between caregivers (Group session only; n = 215)	63·7 (137)

### Study design

We assessed the effectiveness of *Aalana Palana* using a quasi-experimental non-randomized post-test only design embedded within a real-world implementation program. This design was considered as the only feasible method to evaluate the effectiveness of the *Aalana Palana* intervention. Finding from this study could inform more robust designs, i.e., randomised control trials in the future which was beyond the aim and funding remits of this program.

The study started in July 2023, i.e., 19 months after the commencement of implementation. All caregivers of children below 3 years of age residing in the catchment area of the 30 AWCs where *Aalana Palana* was implemented were contacted for participation in the study. A household level mapping was done to identify these eligible caregivers. Participants who gave consent were classified into two groups based on self-reported exposure to the intervention: (i) exposed group included all eligible participants who reported seeing at least one *Aalana Palana* video in the three months preceding data collection, and (ii) unexposed group included participants who did not report seeing any *Aalana Palana* video in the same duration. We decided on a 3-month period to reduce recall bias. The completed Transparent Reporting of Evaluations with Non-randomized Designs (TREND) checklist can be seen in S1 File.

### Measures

We collected socio-demographic characterises and details on exposure to Aalana Palana from all participants. The primary outcome to measure child development was the Caregiver Reported Early Development Instruments (CREDI) [20], a caregiver-reported population-level measure of early childhood development for children aged 0–36 months. The tool consists of four domains – motor, language, cognitive and socio-emotional. We used the long-form which comprises 108 items. With permission from the publishers, the Sangath team translated the CREDI in Telugu. The Telugu version of the form can be found on their website (https://www.dropbox.com/scl/fo/x46wvsf3rno8nrthuvbdy/ALuNPVKWqHBLMQixeRWSaDw/Telugu?dl=0&rlkey=1y0vqdmmvftprlf57pdyppfjj&subfolder_nav_tracking=1). We also used the Family Care Indicators (FCI) [21], developed by UNICEF, which is a caregiver report of the home environment. Adapted from the Home Observation for Measurement of the Environment (HOME) inventory, it focuses on caregiving practices that support early child development in children up to five years. The FCI uses yes/no questions to evaluate sources of play materials, variety of play materials, and play activities. The Infant and Young Child Feeding (IYCF) framework [22] was used to evaluate child nutrition by collecting data on feeding practices critical for growth and development in children under two years, focusing on breastfeeding and complementary feeding adequacy. We used the IYCF data to derive a Minimum Acceptable Diet (MAD) score which is defined as the percentage of children 6–23 months of age who consumed meals at an appropriate frequency and in a sufficient variety to meet their energy and nutrient needs during the previous day.

### Data collection

Data were collected between 10 July 2023 and 01 January 2024 by two teams. Team A consented participants and collected socio-demographic and intervention exposure data while Team B, who were blinded to the exposure data, conducted outcome assessments. The blinded list of consented participants identified by Team A was shared with Team B who visited the households three-to-four weeks post consenting. Teams were trained by senior investigators of *Aalana Palana* and a developmental psychologist with over 20 years of experience proficient in the local language who also led the translation of the CREDI. For the outcome measures classroom trainings were followed by practice assessments where each assessor completed at least five assessments with caregivers and children under supervision.

### Data analysis

All analyses were done using Stata 15 (StataCorp LLC: College Station, Texas, USA) and SPSS 27. For the implementation data, we calculated percentages for characteristics and quality of sessions delivered by AWWs. Descriptive statistics, including means, standard deviations (SD), and percentages, were calculated to summarize the characteristics of the participants, exposure and child development outcomes. All socio-demographic data were compared between exposed and unexposed groups using chi square tests for categorical variables and t test for continuous variables. Each of the 17 FCI items were analysed by estimating percentages of positive responses and all items summed to make a total FCI score [23]. CREDI scores were transformed into z scores as per CREDI scoring norms. We assessed the effectiveness of *Aalana Palana* using regression for the CREDI after controlling for child age, child sex, maternal education, FCI and AWC registration. Further, we did a sensitivity analysis for caregivers registered at AWCs to control for possible independent effects of engaging with the AWC. Logistic regression was used to look at the difference between exposed and unexposed groups for FCI and IYCF. We also conducted moderation analysis to assess whether child sex, child age, and maternal education moderated the effectiveness of *Aalana Palana* on CREDI. Mediation of the effect of the intervention on CREDI through FCI was assessed using Structural Equation Model (SEM) analysis. The minimal anonymized can be found in S1 Data.

#### Financial disclosure statement.

This study was funded by the UNICEF office of Andhra Pradesh, Karnataka and Telangana (Grant no.: IND/PCA20231309/SPD20232091-amd/1). The funder had no role in study design, data collection and analysis, decision to publish, or preparation of the manuscript. RR, GL, DJ, and GD received salaries under this grant during the program implementation.

#### Registration.

This evaluation was registered on ClinicalTrials.gov on 8 June 2025 (Registration numbers: NCT07004608; https://clinicaltrials.gov/study/NCT07004608). Aalana Palana was conceptualised as an implementation program embedded within existing government care systems, not a conventional clinical trial. All necessary ethical approvals for this were sought prior to research data collection; however, registration in an international trials registry, i.e., ClinicalTrials.gov, was not considered necessary. Following feedback during the manuscript review process by journal editorial teams, the program was retrospectively registered. Additionally, the authors confirm that all ongoing and related evaluations for this intervention are also registered.

## Results

### Implementation of Aalana Palana

*Aalana Palana* implementation results can be seen in Table 1. A total of 361 sessions delivered by AWWs were observed by the supervisory team, of which 59·6% (n = 215) were group sessions with an average 4 attendees per session (SD = 1·7, Range 2–11). Session duration varied between 10–30 minutes. Appropriate presentation and navigation of videos were observed in majority of the sessions. Other than demonstrations, most other recommend practices of delivering session including optimal verbal and non-verbal communication and getting support from family members were adopted. Adhering to the intended implementation process of pausing, AWWs paused the *Aalana Palana* video to initiate discussions in 77·6% sessions, addressed concerns on child development raised by caregivers in 80·9% sessions and used some positive gestures to appreciate caregivers when they said something right in 52·9% sessions.

### Participant (caregivers) characteristics

**Fig 1:**
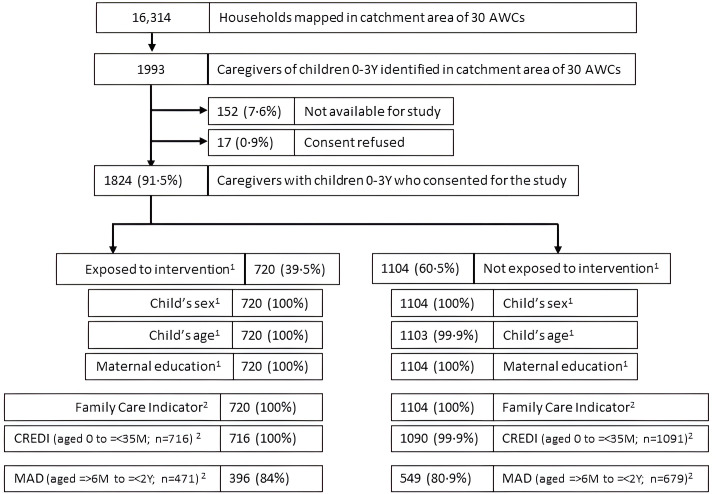
Flow diagram of participant recruitment & data collection.

Details of participant recruitment and exposure to the intervention are reported in Fig 1. 16,314 households were mapped in the catchment area of the 30 AWCs. 1993 caregivers of children < 3 years were identified, of which 91·5% (n = 1824) consented for the study. 39·5% (n = 720) of these caregivers reported having seen an *Aalana Palana* video in the three months preceding the survey; socio-demographic characteristics and FCI were collected for all of them as were CREDI assessments for all children aged 0 to =<35 months in this group (n = 716). For the 60·5% (n = 1104) of caregivers who did not report exposure to *Aalana Palana,* similar data collection rates were achieved except for one child whose CREDI could not be assessed. Data on MAD was collected on the subset of 945 children aged =>6M to =<2Y; data completion for exposed group was 84% (n = 396) and unexposed group 80·9% (n = 549).

The mean child age was 16·3 months (SD = 9·2) and female constituted 51·1% of the sample. There was no difference in child sex and maternal education between the groups. Child age was slightly lower in the exposed group (p = 0·073). Registration at AWCs also differed between exposed and unexposed groups; percentage of caregivers registered was higher in exposed group (χ² = 249·75, = p < 0·001). See Table 2.

**Table 2:** Sample characteristics.

	Exposed	Unexposed		
	% (n)	% (n)		
Child’s Age			***t***	**p**
(Mean, SD in months)	9.2 (16.8)	9.5 (16.0)	-1.797	0.073
Child’s sex			***χ*** ^***2***^	***p***
Girl	51.0 (367)	51.2 (565)	0.007	0.932
Boy	49.0 (353)	48.8 (539)		
Education of mother				
Illiterate & Primary	16.8 (121)	14.9 (164)	3.98	0.264
Middle & High School	26.5 (191)	26.0 (287)		
Post-high school diploma, graduation, and other professional degrees	56.4 (406)	58.2 (643)		
Don’t know/can’t say	0.3 (2)	0.9 (10)		
Registered at AWC	99.4 (716)	70.2 (775)	249.75	<0.001

### Effectiveness of Aalana Palana intervention

Results for the regression model examining the effect of *Aalana Palana* on CREDI are presented in Table 3. Exposure to *Aalana Palana* improved overall CREDI score by 0·16 SD (*p* < 0·001). The full model including covariates explained 32% variance in overall CREDI scores. Exposure to *Aalana Palana* also improved scores of cognitive domain by 0·42 SD (*p* < 0·001), socio-emotional by 0·33 SD (*p* < 0·001), language by 0·18 SD (*p* < 0·001), and motor by 0·06 SD (*p* = 0·005). Similar effects were observed in sub-group analysis of caregivers registered at the AWC (see S1 Table).

**Table 3:** Effectiveness of Aalana Palana exposure with child development outcomes (CREDI overall and domain scores) in children aged 0-3 years (n = 1794).

CREDI scores	Stand. β	t	Adjusted. R^2#^	*F (28, 1765)*
CREDI overall score	0·16	7·47**	0·32	30·74**
*Cognitive*	0·42	20·32**	0·37	38·27**
*Language*	0·18	8·07**	0·26	23·51**
*Motor*	0·06	2·79*	0·27	24·15**
*Socio-emotional*	0·33	15·02**	0·28	25·99**

^#^For full models including child age, child sex, maternal education, FCI items and registration at AWC;

* p < 0·01; **p < 0·001.

The effects of *Aalana Palana* on FCI and MAD are presented in Table 4. Exposure to *Aalana Palana* was associated with increased odds of most items aligned with responsive parenting. MAD did not differ between exposed and unexposed groups.

**Table 4:** Descriptive statistics and logistic regression for FCI and MAD.

	Exposed (n = 718)	Unexposed (n = 1093)	OR (95% CI)	z-value	p
Home-made toys	6·5 (47)	6·8 (74)	0·93 (0·62, 1·40)	-0·32	0·746
Toys bought from store	92·9 (667)	90·7 (991)	1·06 (0·71, 1·59)	0·31	0·755
Household objects (such as bowls or pots)	85 (610)	79 (863)	1·32 (0·97, 1·80)	1·74	0·081
Objects found outside (sticks, rocks, animal shells or leaves)	73·3 (526)	67·1 (733)	1·10 (0·83, 1·46)	0·66	0·512
Play with things which make/play music	71·3 (512)	64·4 (704)	1·31 (1·04, 1·64)	2·31	0·021
Play with things for drawing/writing	50·8 (365)	45·1 (493)	1·08 (0·84, 1·39)	0·62	0·537
Play with picture books for children (not school books)	40 (287)	31·1 (340)	1·41 (1·13, 1·77)	3·01	0·003
Play with things meant for stacking, constructing, or building	40·9 (294)	32·9 (360)	1·30 (1·02, 1·66)	2·15	0·032
Play with things for moving around (balls, bats, etc.)	66·7 (479)	63·5 (694)	1·05 (0·80, 1·38)	0·35	0·723
Play with toys for learning shapes and colours (circle, square, triangle)	27·9 (200)	20·8 (227)	1·39 (1·08, 1·79)	2·53	0·011
Play with things for pretending (dolls, tea-set, etc.)	61·6 (442)	57·4 (627)	1·06 (0·80, 1·40)	0·40	0·690
Read books or look at picture books with child	34·5 (248)	22·9 (250)	2·07 (1·63, 2·64)	5·90	<0·001
Tell stories to child	45·3 (325)	38·1 (416)	1·32 (1·07, 1·62)	2·58	0·010
Sing songs with child	70·2 (504)	59·5 (650)	1·60 (1·28, 1·99)	4·11	<0·001
Play with the child with toys	94·7 (680)	86·3 (943)	2·86 (1·93, 4·24)	5·23	<0·001
Take child outside home place	87·6 (629)	82·6 (903)	1·41 (1·03, 1·92)	2·17	0·030
Spend time with child in naming things, counting, drawing	41·9 (301)	35·3 (386)	1·37 (1·09, 1·72)	2·74	0·006
Availability of books	33·6 (241)	25·5. (279)	1·42 (1·12, 1·81)	2·86	0·004
	**(n = 395)**	**(n = 546)**			
Minimum Acceptable Diet	45·3 (179)	37·4 (204)	1·28 (0·95, 1·73	1·62	0·104

**Fig 2:**
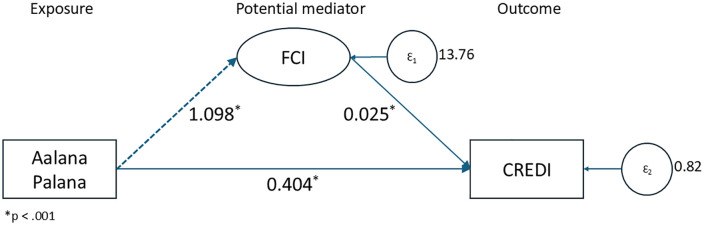
Mediation analysis.

Results for mediation analysis on the effect of *Aalana Palana* on CREDI through FCI are shown in Table 5 and Fig 2; FCI mediated the effect of the intervention on CREDI (b = 0·03, p < 0·001).

**Table 5:** Mediation of the effects of the Aalana Palana intervention on child development (CREDI).

	Coef.	z	95% CI
Direct Effects			
AP → FCI	1·098*	6·15	0·748 - 1·447
FCI → CREDI	0·025*	4·37	0·014 - 0·036
AP → CREDI	0·404*	9·17	0·318 - 0·490
Indirect Effect			
AP → FCI → CREDI	0·027		0·014 - 0·044
Total Effect			
AP → CREDI	0·431		0·035 - 0·052

*p < 0·001.

Results of the moderation analysis can be seen in Table 6. Child sex (β = 0·068, p = 0·364) and maternal education (β = -0·148, p = 0·063) did not show any moderating effects. Effect of *Aalana Palana* on CREDI was moderated by age (β = 0·238, p < 0·001) with younger children showing better overall CREDI scores.

**Table 6:** Moderation of the effectiveness of Aalana Palana.

Effect on CREDI	n	Stand. β	t	p	Adjusted R^2^	*F*
AP x Child’s Sex	1806	0·068	0·91	0·364	0·053	34·40 (3, 1802)
AP x Maternal Education	1794	-0·148	-1·86	0·063	0·056	27·77 (4, 1789)
AP x Age (in months)	1806	0·238	10·06	<0·001	0·095	63·96 (3, 1802)
>=13 to < 25		-0·269	-10·70	<0·001		
>=25 to <36 months		-0·254	-9·77	<0·001		

## Discussion

This study aimed to evaluate the effectiveness of *Aalana Palana*, a facilitated video-viewing parenting intervention on home environment, feeding practices, and child development outcomes. Notably, *Aalana Palana,* which focuses on responsive and sensitive parenting, was integrated within the ICDS, and was delivered through group and individual sessions by AWWs in community settings. We observed high levels of fidelity of the delivery of the intervention over a 19-month implementation period, its effectiveness on all hypothesized outcomes, mediation of the intervention effects on child development by changes in home environment and, greater effects in younger children which aligns with existing evidence on early interventions [24,25]. This study adds to the scarce evidence on how established parenting interventions which have been rigorously tested in controlled clinical trials [26] can be embedded in and scaled up with routine maternal and childcare services. Although, responsive caregiving has emerged as the central mechanism for behaviour change, around which other nurturing care domains can be scaffolded, greater attention to contextualised, integrated implementation approaches is required to strengthen early childhood development programming at scale [27]. Thus, the strength of the study lies in assessing effectiveness in a real-world context, as we deployed existing government AWWs to deliver the intervention as part of their routine maternal and child care services, enhancing the generalizability of our findings.

We attribute these favourable findings to a number of key design features of the implementation of the intervention. All trainings and ongoing supportive supervision were modelled around existing ICDS systems and pragmatic, easy to integrate processes. This is demonstrated by a streamlined training schedule, no additional burden of reporting for AWWs, monitoring *Aalana Palana* exposure through existing record keeping, and deploying an objective observation tool to assess the quality of sessions. Based on these observations it could be concluded that AWWs were able to include *Aalana Palana* within their routine work, deliver the intervention with high levels of fidelity, and gain proficiency in using the videos while conducting group sessions which aligns with evidence of the impact of delivering group-based parenting interventions on child development outcomes [4]. However, we also observed that some of the key strategies of demonstration which can effectively support caregivers to learn new skills [28] were not optimally used by the AWWs. A sufficiently long period of delivery enhanced the likelihood of detecting an effect on child development outcomes which modify gradually over time.

Exposure to *Aalana Palana* showed improvements in overall CREDI scores and particularly in the cognitive and socio-emotional domains which aligns with the intervention’s focus on responsive and sensitive caregiving practices. This is in line with existing evidence that underscores the importance of caregiver-child interactions in fostering early cognitive and socio-emotional development [29]. However, effect on motor development was small, reflecting the absence of targeted messaging on physical activities in the intervention content and suggests an area for future video content enhancement. Aligned with the theory of change of the intervention, we observed mediation by the proximal environment of children on child development outcomes [30] and, aligned with developmental theories of brain plasticity [31], a greater effect on younger children, when the brain is thought to be more sensitive to environmental influences.

The study has several limitations. We used a non-randomized design which is implicit in the fact that this was a real-world implementation study. Unavailability of baseline data meant we could not estimate changes from baseline or potential confounders; however, all our effectiveness analyses adjusted for child sex, age and maternal education. Although data was collected from 1824 participants, they were limited to the catchment area of 30 AWCs. Control group participants were not recruited before the start of intervention and exposure status was ascertained based on participant recall of having seen any *Aalana Palana* video in the 3 months preceding the survey. Number of videos seen in this period were not monitored. This might not fully explain actual exposure to the intervention or account for previous exposure to *Aalana Palana* among the unexposed group. Notably, the study did not collect information on gestational age, child anthropometry, health service utilization and adversities that have proven to impact child development outcomes [32], though there is no reason why these should differ between the two groups. Importantly, the team of assessors deployed to collect the outcome data were separate from those collecting exposure data, reducing the risk of observer bias [33].

The findings highlight several implications for the adaptation and scale-up of *Aalana Palana*. These proof-of-concept findings can inform future studies employing more rigorous experimental or longitudinal designs. The videos showed real-life examples of caregiver-child interactions that were culturally and contextually relevant which increased acceptability among AWWs and supported improvements in children’s socio-emotional and cognitive development. The planned pauses in the videos encouraged discussion and reflection. These features can be scaled and adapted to other settings to improve acceptability, uptake, and effectiveness. *Aalana Palana* has now been mandated as one of the community-based interventions aligned with the ICDS Guidelines for Supplementary Nutrition Programme in the state of Telangana, indicating the buy-in and impact of our study findings.

The core principles of responsive caregiving are generalisable across settings; however, the community-specific visual content limits broader transferability, as these may not reflect the cultural diversity within India. While the underlying behavioural concepts can be retained, nutrition-related messaging would require adaptation for other settings based on local IYCF practices.

Future implementations need to increase representational diversity, incorporate caregiver testimonials, and enhance CBW capacity and motivation to facilitate caregiver-led discussions, shared reflections, demonstrations, and scaffolding. Also, studies should consider a priori evaluation, deploying a randomized design, estimating effectiveness and cost-effectiveness, and the mechanisms of action to guide the larger scale-up of the intervention.

## Supporting information

S1 Text Blueprint and structure of *Aalana Palana* intervention videos. (DOCX)

S1 File TREND_Checklist. TREND statement.
(DOC)

S1 Data Anonymized minimal dataset and data dictionary used for study analysis. (XLSM)

S1 Table Results of sub-group analysis for caregivers registered at the AWC (n = 1471). (DOCX)
