# Supplementary material for: The effectiveness of a community-based video-facilitated parenting intervention for child development integrated into routine maternal and child care services in India
Source: PLOS Glob Public Health. 2026 Mar 2;6(3):e0005434. doi: 10.1371/journal.pgph.0005434 (PMC12952612; doi:10.1371/journal.pgph.0005434)
Supplement: S1 Text — (DOCX) [file pgph.0005434.s001.docx]

# S1 Text

# *Aalana Palana* Videos Blueprint

1. **Video 1**
   1. Target audience: Pregnant Women
   2. Key messages:
   - Women should register their pregnancy at the AWC​
   - It is important to go for a minimum of four antenatal checkups with a doctor during pregnancy; at 12 weeks, 14-26 weeks, 28-34 weeks of pregnancy and after 36 weeks of pregnancy​
   - It is crucial to have a balanced diet consisting of include nutritious food and 8-12 glasses of water daily during this time​
   - It is important to take iron and calcium tablets daily​
   - Exposure to cigarettes and alcohol should be avoided during this time​
   - It is important for the pregnant woman to rest and get help with housework​
   - Light exercises are advised for physical and mental well-being​
   - Preparing for delivery in advance helps ensure that everything will go smoothly on the day.
   - It is important to feed your newborn child with colostrum within 1 hour of birth to fight infections and help in physical and mental development of her child.
   - Breastfeeding in the first golden hour helps the milk to come in, reduces bleeding and gives your baby their first dose of immunity
2. **Video 2**
   1. Target Audience: Caregivers of children aged 1-3 months
   2. Key messages:
      - It is important to exclusively breastfeed until the child is 6 months of age
      - One should feed the baby as frequently as required, which may be up to 8-10 times a day
      - It is important to hug, caress, look, smile, coo & talk to the baby to make them feel safe & loved
      - Do not wrap your baby tightly in clothing for long periods of time. They need to be able to move & touch people & things
      - Playing with colorful toys & those that make noise helps to stimulate the baby’s vision & hearing
      - Massaging baby with natural oils using firm but gentle strokes supports the baby to feel secure, may improve their digestion & promotes better sleep
      - It is of primary importance to build a strong caregiver-baby relationship in this time
3. **Video 3**
   1. Target Audience: Caregivers of children aged 4-6 months
   2. Key messages:
      - It is important to exclusively breastfeed until the child is 6 months of age
      - After completing 6 months, when the child slowly starts eating, it is okay if the child plays with food & makes a mess while they eat, as it helps them learn
      - Babies are now making sounds & doing actions to communicate with caregivers. By copying their sounds, talking back about things they may be interested in caregivers can help children learn early language
      - Learning in babies can be encouraged by allowing them to play with different objects around the house & letting them manipulate these by grabbing, dropping & mouthing them.
      - Ensure that objects are clean & big enough so that the child will not choke on them.
      - Playing with the child & singing to them helps them feel happy, calm & safe
4. **Video 4**
   1. Target audience: Caregivers of children aged 7-9 months
   2. Key messages:
      - Children can now gradually start eating different varieties of foods
      - Mealtimes should be hygienic by washing hands before cooking and eating
      - Make mealtimes fun if children are allowed to explore their food
      - Children start to understand their name and many other words in this age, even if they haven’t started speaking yet so family members can communicate with them to encourage them to speak
      - Since children can now differentiate strangers from known people, introducing them to new people can help them make friends
      - Exploration should be encouraged even if it involves making noise as it helps children learn!
      - Showing children pictures of different objects and body parts helps them learn new words
5. **Video 5**
   1. Target audience: Caregivers of children aged 10-12 months
   2. Key messages:
      - As the child starts eating different varieties of food, they might express their dislike for specific food, which should be permitted
      - It is important to keep the child’s surroundings neat and clean to prevent any form of infection
      - Eating on their own makes the child feel independent​
      - Children can differentiate strangers from known people. They also get upset when a familiar caregiver leaves but can be made can be comforted if reassured of a safe return or left with a familiar object ​
      - Everyday activities like sorting and games such as those involving hiding and looking for objects promote exploration and learning
6. **Video 6**
   1. Target audience: Caregivers of children aged 13-15 months
   2. Key messages:
      - The child should eat various foods rich in Vitamin A which are good for their vision. Parents need to provide the child with foods that give them energy.
      - The child should eat with the rest of the family. By doing this, the child will observe & imitate the rest of the family eating behaviours
      - At this age, the child will understand words & asks for things by pointing towards them. The caregiver should interact with the child; this improves the communication & language skills of the child
      - Encouraging the child to walk on their own by asking them to bring the toys to the caregiver, this will help in developing the child’s muscles
7. **Video 7**
   1. Target audience: Caregivers of children aged 16-18 months
   2. Key messages:
      - Every month on the 1st the child should be taken to AWC for measuring their height & weight
      - Balamrutham is given in AWC every month. The ingredients in the Balamrutham are highly nutritious
      - Encouraging the child’s independence. The child should know that it is ok to make a mess.
      - The child starts to explore the house and starts playing with the toys by banging, hitting or shaking them. As the child starts exploring, tell them the name of the objects, share some stories related to the objects, persons & places
      - Introducing the child to new people & environment. The child should feel safe & secure in presence of others. The caregiver can help the interaction by introducing the child to neighbors & their children to make them feel comfortable
      - The child will be able to identify body parts at this age. By making them understand the differences in body parts of others, the child understands the concept of “me” & “others”.
      - The child enjoys the action of copying others action within the house. This helps with the child’s imagination & also their personality development
8. **Video 8**
   1. Target audience: Caregivers of children aged 19-21 months
   2. Key messages:
      - Introducing variety of foods in the child’s meal can help meet their nutrition requirements.
      - Since the child may have started holding objects in hand, encourage them to write in creative ways. For example, the caregiver can ask for child’s to water the plants which can help in child’s understanding about plants.
      - Children love listening & learning from caregivers. The caregiver can narrate the stories by acting & singing them. This helps the child to listen, interact & learn language. By dancing along with the child the caregiver helps in the development of the child’s muscles.
      - Children like to look in the mirror. By doing this, the child understands that the others look different around them. The caregiver can show them their pictures & make them understand that they are different from others.
      - Children while playing & interacting with other children learn new words & helps form friendships. But the caregivers should be present with the children to reassure & make them feel comfortable while playing.
9. **Video 9**
   1. Target audience: Caregivers of children aged 22-24 months
   2. Key messages:
      - The child can now eat independently. The caregiver can encourage this by giving the child a separate bowl & spoon. By making the child sit with family members during meals, the child starts imitating family members & learns how to eat without spilling.
      - Every month on the 1st, the child should be taken to AWC to check their height, weight & Mid Upper Arm Circumference (MUAC). This checkup ensures that the child is growing properly.
      - The child starts to select what they want to play with. Caregivers & children can make toys which the child wants to play at home. This increases the interaction between them & helps in child’s learning.
      - Caregiver should involve in fun activities like hiding the objects & then should encourage the child to find hidden objects, making the child understand that objects are present & do not disappear even if they are out of sight.
      - Teaching concepts by playing with the children is the best way to make them understand. This can also be done if the child plays with other children.
      - By making the child play with objects, it helps them understand about concepts of different sizes, shapes & colours.
10. **Video 10**
    1. Target audience: Caregivers of children aged 25-60 months
    2. Key messages:
       - The caregiver should provide 3 full meals along with snacks 2-3 times a day.
       - The child now will start to eat food by themselves but it is important the caregivers observe the child while eating.
       - Children often like to play with dough. The caregiver can use dough to teach the child how to make shapes, numbers, animals and other things to refine their skills of fine movement.
       - Giving the child a slate or book & a chalk or crayon, encourages the child to draw.
       - Encouraging the child to play with other children helps in their overall physical growth & also build early relationships. Children understand the concept of “mine” while playing with their toys.
       - Caregiver can increase the child’s communication skills by asking them questions or reading stories with them. The child while speaking makes many mistakes but the caregiver should encourage the child to continue talking.
       - Children may exhibit various behaviors while growing up. This increases when they are hungry, tired & sleepy. Some behaviors like throwing toys are part of child’s learning.
       - Caregivers should not hit or scold the child. They should try to remain calm and promote good behavior by praising the child
11. **Video 11**
    1. Target audience: Family members of children aged (fathers, grandparents and other caregivers)
    2. Key messages:
       - It is important to register the mother and child at the nearest AWC. Since all family members play a role in the child’s development, grandparents and fathers should accompany the mother during regular visits to AWC.
       - All members of a household can help to ensure that the mother gets timely and nutritious meals while she is nursing her child. Eating along with all the family members can help ensure that she is taking care of her nutrition needs
       - A mother and baby should be relaxed and undisturbed especially in the early days when breast feeding, bonding is being established. Family members should support the mother by taking over household chores
       - Fathers can play an active role in their child’s development by spending time and communicating with them.
       - Father and child reading together helps in the development of the child’s language and communication skills
       - Singing to the child is fun and also helps further interactions between grandparents and children
       - Fathers can engage in routine caregiving with the child and use it as an opportunity to bond.
