# Supplementary material for: The effectiveness of a community-based video-facilitated parenting intervention for child development integrated into routine maternal and child care services in India
Source: PLOS Glob Public Health. 2026 Mar 2;6(3):e0005434. doi: 10.1371/journal.pgph.0005434 (PMC12952612; doi:10.1371/journal.pgph.0005434)
Supplement: S1 Table — (DOCX) [file pgph.0005434.s004.docx]

# S1 Table

**Sub-group analysis for caregivers registered at the AWC (n=1471)**

|  | Stand. β | t | p | Adjusted R^2^ | *F* |
| --- | --- | --- | --- | --- | --- |
| CREDI overall score | 0.16 | 7.26 | <0.001 | 0.32 | 26.83 |
| Cognitive | 0.42 | 20.07 | <0.001 | 0.39 | 35.21 |
| Language | 0.18 | 7.89 | <0.001 | 0.27 | 21.22 |
| Motor | 0.06 | 2.69 | 0.01 | 0.27 | 21.62 |
| Socio-emotional | 0.33 | 14.75 | <0.001 | 0.30 | 23.88 |
